# Integrating clinical and economic evidence in clinical guidelines: More needed than ever!

**DOI:** 10.1111/jep.12936

**Published:** 2018-04-26

**Authors:** Saskia Knies, Johan L. Severens, Werner B.F. Brouwer

**Affiliations:** ^1^ National Health Care Institute PO Box 320 1110 AH Diemen The Netherlands; ^2^ Erasmus School of Health Policy & Management Erasmus University Rotterdam Burgemeester Oudlaan 50 3062 PA Rotterdam The Netherlands; ^3^ Institute of Medical Technology Assessment Erasmus University Rotterdam Burgemeester Oudlaan 50 3062 PA Rotterdam The Netherlands

**Keywords:** clinical guidelines, evidence‐based medicine, health economics, health care

## Abstract

**Rationale, aims, and objectives:**

In recent years, several expensive new health technologies have been introduced. The availability of those technologies intensifies the discussion regarding the affordability of these technologies at different decision‐making levels. On the meso level, both hospitals and clinicians are facing budget constraints resulting in a tension to balance between different patients' interests. As such, it is crucial to make optimal use of the available resources. Different strategies are in place to deal with this problem, but decisions on a macro level on what to fund or not can limit the role and freedom of clinicians in their decisions on a micro level. At the same time, without central guidance regarding such decisions, micro level decisions may lead to inequities and undesirable treatment variation between clinicians and hospitals. The challenge is to find instruments that can balance both levels of decision making.

**Discussion:**

Clinicians are becoming increasingly aware that their decisions to spend more resources (like time and budget) on 1 particular patient group reduce the resources available to other patients. Involving clinicians in thinking about the optimal use of limited resources, also in an attempt to bridge the world of economic reasoning and clinical practice, is crucial therefore. We argue that clinical guidelines may prove a clear vehicle for this by including both clinical and economic evidence to support the recommendations made. The development of such guidelines requires cooperation of clinicians, and health economists are cooperating with each other.

**Conclusion:**

The development of clinical guidelines which combine economic and clinical evidence should be stimulated, to balance central guidance and uniformity while maintaining necessary decentralized freedom. This is an opportunity to combine the reality of budgets and opportunity costs with clinical practice. Missing this opportunity risks either variation and inequity or central and necessarily crude measures.

## 1 INTRODUCTION

The affordability of new health technologies, amongst which expensive pharmaceuticals, remains an important issue at different decision‐making levels in health care. In addition to national decision makers (mostly involved in reimbursement decisions), also hospitals and clinicians are directly or indirectly faced with budgetary constraints. As a result, also hospitals and clinicians struggle to balance budget constraints with their patients' interests. Finding this balance is extremely difficult at any level and is high on the political agenda in many countries. Failure to make such choices at a national level and to provide adequate financing of new technologies leads to pressure, choices, and potentially inequities at lower levels of the health care system. This is also true for positive reimbursement decisions at a central level, which may lead to pressure on existing budgets if these do not grow to cover the related (net) expenses.^1^

This tension between all patients' needs and available budget reflects the classical issue that health care systems simply do not have enough resources to provide all patients with every available (potentially) effective clinical intervention.^2^, ^3^ Because the tension between budgetary constraints and patients' demands and needs is unavoidable, it is crucial to make optimal use of the available resources. Countries use different strategies in this context, including decision making based on health technology assessments, price negotiations, and restricting access to expensive pharmaceuticals.^4^ Macro level decisions may give financial access and stimulate equity, but they can also be relatively crude as they tend to make technologies accessible to either all patients, some selection of patients, or none. This does not do justice to the often encountered variation within patient populations and treatment effects, which clinicians observe at the micro level. Surprisingly, these clinicians are not always involved in macro decisions which does not do justice to their important role. On the meso level, clinicians are typically part of the struggle to balance limited resources and individual patients' interests for instance in budget allocations over different specialities. These choices have implications for the technologies offered to patients and may vary between hospitals. Leaving the difficult decisions regarding what to fund or reimburse and for whom up to individual clinicians for each individual patient, or on a micro level, could lead to further inequities and undesirable reatment variation. The challenge is therefore to find a golden middle to balance uniformity at a central level and flexibility at the micro level.

It seems that this search is still ongoing. Clinicians increasingly feel the pressure of limited available resources, whether or not they are responsible for budgets themselves. They appear to be well aware of the fact that their decisions have an impact on the resources available to other patients. Hence, clinicians may consider whether their health care organization, health care system (especially mandatory tax or insurance financed ones), or even society as a whole can afford specific technologies.^5^ Involving clinicians in thinking about the optimal use of limited resources, also in an attempt to bridge the world of budgets and clinical practice, is crucial therefore.

## 2 DISCUSSION

### 2.1 Need for efficient guidelines

Evidence‐based clinical guidelines, for which a rising interest can be noticed, may well prove the best instrument to link the macro and micro level. These guidelines are developed, often by medical professionals associations, as a tool to inform clinicians about the current state of knowledge regarding the benefits and limitations of specific technologies for defined health problems.^6^, ^7^ The increasing complexity of health technologies, a growing desire to increase quality of care and reduce variation, and the necessity to control costs, has led to the growing use of clinical guidelines in the last 3 decades.^8^ Guidelines may thus be used as a means for internal quality assurance by optimizing the actions of individual clinicians, whereby it is important to note that quality is a multidimensional concept.^7^ For instance, the World Health Organization distinguishes 6 dimensions of quality, being: effectiveness, efficiency, accessibility, acceptability/patient‐centered, equity, and safety.^9^ Clinical guidelines can contribute to improve quality of care in different ways, but it can be argued that not all aspects of quality are equally relevant in the context of guidelines.^7^, ^10^ In general, clinical guidelines try to inform clinicians about the potential effectiveness and safety of a specific technology for a defined health problem. Until now, the majority of clinical guidelines have paid limited attention on efficiency of care as a dimension of quality.^7^

Critical questions can be raised on whether clinicians have to deal with economic arguments in individual patient decisions or whether clinical guidelines should incorporate economic arguments. Dutch findings indicate that clinicians think that it is possible to take cost‐effectiveness data into account in clinical practice; however, they do not want to make the decision in their consulting room without further guidance (and backing). Incorporating cost‐effectiveness data in clinical guidelines is therefore considered as the way forward.^11^ This accords with the message of optimizing the allocation of scarce health care resources by informing decisions using economic evidence, as health economists try to bring across. In order to do so, all other things equal, health care systems could prioritize technologies that result in the highest health gain per monetary unit spent. In appraising these technologies, other aspects (and health system goals) like equity can be included as well. But by prioritizing clinical practices that are the most cost‐effective, guidelines can help in the pursuit of optimizing population health from a given budget. As clinical guidelines aim to advice on which treatments to give under which circumstances, guidelines are very suitable for promoting cost‐effective clinical practice on a level of detail that is not encountered in normal central reimbursement decisions.^3^ Such practice acknowledges that health technologies are not effective or cost‐effective per se, but only in the appropriate target group and circumstances.

Ignoring economic evidence when developing clinical guidelines could result in treatment recommendations that do not represent a cost‐effective use of health technologies and may therefore ultimately be harmful for the use of other technologies, patients, and population health. Moreover, failure to address this pressing matter through the here proposed route results in decisions being made at other levels, given that resources remain too scarce to treat all patients to the best possible extent. Then decisions may be either crude (at macro levels) or varying between hospitals (meso level) or clinicians (micro level). Without considering economic evidence, guidelines may add to the tension between available recourses on the one hand, and treatment options and patients demands on the other. Ideally, guidelines indicate how to offer optimal care taking the available resources into consideration, by incorporating cost‐effectiveness data in the recommendations stated in clinical guidelines.^12^ Guidelines could in this way increase quality of care and ensure an efficient allocation of resources at the same time.^7^, ^8^

### 2.2 Experience in England and the Netherlands

Is there any evidence that this can work? Both England and Wales and the Netherlands have some positive experience including economic evidence in clinical guidelines. In England and Wales, cost‐effectiveness is considered at every stage of the development process of new clinical guidelines. The development of a guideline starts with the development of the scope. Next, the guideline itself is development in which a systematic literature review is conducted and, if necessary, additional cost‐effectiveness studies are performed. Third, the recommendations are developed for each topic in the guideline based on the earlier collected clinical and economic evidence. Finally, the guideline is drafted, and a consultation follows by asking stakeholders their opinion, after which the guideline is implemented.^3^ A problem arises when limited clinical evidence is available, but with substantial cost differences exist. In this case, the choice has to be made what option to recommend.^3^ From experience in the Netherlands, we can learn that it is important to understand the viewpoint from various professions, and the complementarity in the overall aim of improving population health. In addition, the members and (especially) the chair of the guideline development group should be convinced of the added value of incorporating health economic evidence.^13^ The role of clinicians in this process of incorporating cost‐effectiveness data in their guidelines is crucial as their clinical knowledge has to be combined with economic arguments.

The experience from the Netherlands and England and Wales shows that combining clinical and economic evidence is possible, but that it is important to consider economic evidence in every stage of developing clinical guidelines. Such guidelines stimulate cost‐effective care by limiting the use of health technologies to those situations in which they can be considered to offer value for money. The focus on the value of the care delivered is in line with recent developments towards value‐based health care. One of the steps taken to influence health care providers in improving the value of health care is to create guidelines identifying best practices and value for money.^14^ Putting limitations on the use of health technologies using economic evidence is not based on the wish to save money but to be able to create more value: save more lives, improve quality of life, and further improve overall population health using the available resources as efficiently as possible.

## 3 CONCLUSION

Developing guidelines based on clinical evidence, incorporating economic data is impossible without an intensive collaboration between clinicians and health economists. Crude choices and undesirable treatment variation may be avoided by, next to clinical evidence consistently including economic evidence in clinical guidelines. Ideally, national decision‐making bodies set the boundaries and principles for developing such clinical guidelines, after which clinicians specify the guidance with inputs from health economists. Next to that, sufficient resources should be available to be able to implement the recommendations in guidelines in daily practice. In other words, the financial streams need to enable the actors to provide the care that society deems necessary, effective, and cost‐effective.

While clinicians may see cost‐effectiveness as something alien, threatening rather than improving health, the opposite may be true. Joining forces allows for tailor‐made, patient centered, and cost‐effective clinical guidelines, contributing to population health: joint input from both professions is more needed than ever!
